# Modelling prevalence and incidence of fibrosis and pleural plaques in asbestos-exposed populations for screening and follow-up: a cross-sectional study

**DOI:** 10.1186/1476-069X-7-30

**Published:** 2008-06-20

**Authors:** Christophe Paris, Aurélie Martin, Marc Letourneux, Pascal Wild

**Affiliations:** 1 Inserm ERI-11, Assessment and prevention of occupational and environmental risks Medical School, 9 av de la Forêt de Haye – BP 184, 54505 Vandoeuvre-les-Nancy Cedex, France; 2 Nancy-University, 1 rue Lyautey, 54000 Nancy, France; 3 Occupational Diseases Department, University Hospital, Avenue de la cote de Nacre, 14000 CAEN, France; 4 Département Epidémiologie en Entreprises, Rue du Morvan, CS 60027,54519 Vandoeuvre Les Nancy Cedex, France

## Abstract

**Background:**

CT-Scan is currently under assessment for the screening of asbestos-related diseases. However, to date no consensus exists as to how to select high-risk asbestos-exposed populations suitable for such screening programs. The objective of this study is to select the most relevant exposure variables for the prediction of pleural plaques and asbestosis in order to guide clinicians in their use of CT-Scan.

**Methods:**

A screening program of non malignant asbestos-related diseases by CT-scan was conducted among asbestos-exposed volunteers in France. Precise assessments of asbestos exposure were obtained by occupational hygiene measurements and a job-exposure matrix. Several parameters were calculated (time since first exposure, duration, intensity and cumulative exposure to asbestos). Predictive parameters of prevalence and incidence were then estimated by standard logistic and a complementary log-log regression models.

**Results:**

1011 subjects were recruited in this screening program among them 474 (46.9%) presented with pleural plaques and 61 (6.0%) with interstitial changes compatible with asbestosis on CT-scan. Time since first exposure (p < 0.0001) and either cumulative or mean exposure (p < 0.0001) showed independent associations with both pleural plaques and asbestosis prevalence and pleural plaques incidence. Modelling incidence of pleural plaques showed a 0.8% to 2.4% yearly increase for a mean exposure of 1 f/ml.

**Conclusion:**

Our findings confirmed the role played by time since first exposure and dose but not duration in asbestos-related diseases. We recommend to include these parameters in high-risk populations suitable for screening of these diseases. Short-periodicity of survey of pleural plaques by CT-Scan seemed not to be warranted.

## Background

CT-scan is without contest the reference tool for diagnosis of asbestos-related diseases as this technique is both more sensitive and more specific than chest X-rays [1]. An international consensus conference [2] has recommended the use of CT-scan in exposed population for clinical individual evaluation or research purposes with respect to pleural plaques and asbestosis. Several countries, who develop thoughts about specific compensation funds for non malignant asbestos related diseases such as the American Senate or the French Law, recommend the use of CT-scan for the diagnosis of such diseases. The prevalence of both pleural plaques and asbestosis is associated with time since first exposure to asbestos, intensity level, duration or cumulative exposure to asbestos depending on the studies [1,3,4], but these criteria remain entangled. Moreover, the majority of these published studies are based on chest X-ray data, resulting in difficulties and imprecision for the estimation of dose-response relationships for asbestos-related diseases. Thus in the few papers predicting the prevalence and incidence of pleural plaques [5] or asbestosis [6], none used CT-scan data and the shape of the dose-response relationships remains uncertain [7]. As a result, Begin et *al*. underlines that CT-Scan is not yet a "gold-Standard" despite his higher sensitivity relative to CXR because of absence of clearly established exposure-response relationships [8]

Recommendations for potential applications of CT scan in lung cancer screening among asbestos exposed subjects have also been reported [4]. Among these statements, the need to assess the respective role of known risk factors of lung cancer (latency and cumulative exposure to asbestos, smoking characteristics, age, asbestos related radiographic findings) was highlighted in order to define populations suitable for such screening programs. Since then several authors [9-11] have recently proposed CT-scan lung cancer screening programs in exposed to asbestos populations with empiric definition of "high risk" group. Accordingly, a widespread CT-Scan screening of occupationally asbestos-exposed populations could become a major public health option, only if it is possible to define reliably high-risk groups.

The present paper presents the results of a modelling based on a large population with detailed asbestos exposure information and systematic CT-scans. The aim was to determine which exposure parameters are most useful to the clinicians in the selection of asbestos-exposed subjects to be submitted to a CT-Scan as part of a screening exercise.

## Population and methods

### Eligibility criteria

A screening program for asbestos-related diseases was instated in 1991 in Normandy (France). We included in this study volunteers subjects over 50 years, including retirees whatever their ages, from specific plants well known to have past heavy use of asbestos, mainly asbestos textile and friction materials fabrication, insulation and energy production The program began by the most exposed workers (as a result the older ones), and was then progressively extended to the more recently exposed subjects. Detailed methods and preliminary reports were previously published [12,13] concerning this program.

All subjects had to be free of previous involvement in systematic HRCT screening campaigns in their companies, and had to be not known to carry asbestos-related diseases prior to the time of inclusion. Retired subjects were recruited either by mail from their former employers or through local information meetings. Active workers were informed by their occupational physicians. All included subjects gave their written informed consent. All investigations were performed in four hospitals located in Rouen, Le Havre, Caen and Flers.

### Clinical and Occupational data

All subjects underwent a standard interview in order to obtain occupational, medical and smoking histories. For occupations implying asbestos exposure in one or several companies, dates of hire and end of assignments as well as durations of exposure were recorded. For some subjects who had worked in an asbestos textile and friction material plant, a quantitative assessment of occupational exposure was obtained using a specific Job Exposure Matrix (JEM) elaborated from airborne measurements collected annually between 1959 and 1999 in the various workshops of the plant. For all other subjects, estimation of asbestos-exposure level associated with each job was assessed using published airborne measurements available in the French Database Evalutil [14] according to the calendar period of exposure and the reported usual tasks.

For all subjects, a cumulative exposure index (CEI) was then calculated and was expressed in fibers/ml.years. It was obtained by summing over all job positions held, with reference to the occupational calendar established in the interview, products of typical job exposure level (in fibers/ml) by job duration (in years). An average exposure index was calculated by dividing the CEI by the duration of exposure. Time since first exposure to asbestos, duration of exposure (years), cumulated exposure index and the average exposure index were used in the statistical analysis as asbestos exposures descriptors.

### Diagnosis of pleural plaques and asbestosis from imaging

Conditions of CT scan were previously described in detail [13]. Briefly, all subjects underwent a conventional or spiral CT-scan without contrast material depending on the date of inclusion. In order to minimize difference between the two generations of CT-Scan, only incremental CT-Scan with joined centimetric slices of complete thorax were kept. However, in both cases, all examinations included HRCT slices. The two generation of CT Scan differed by the number of HR slices as the first kind included at least six high-resolution millimetric sections in prone position and full inspiration, five of which were equally spaced between the carina of trachea and the bottom part of the costophrenic angles, whereas the remaining section was half way between the carina of trachea and the extreme pulmonary apices. For spiral CT-Scan, acquisitions slices were conducted in prone position only if the first sequence showed interstitial abnormalities.

Reading was carried out using a standard grid describing the features of (a) asbestos-related pulmonary fibrosis lesions (i.e., interlobular septal thickening, intralobular lines, honeycombing, subpleural curvilinear lines and ground-glass opacity), and (b) pleural fibrosis lesions (i.e., pleural plaques and diffuse pleural thickening), as well as (c) the pulmonary opacities associated with pleural changes (i.e., parenchymal bands and rounded atelectasis), as described by several authors [15,16]. In particular, pleural plaques were defined as discrete, dense, pleural linear structures, which may have a smooth or nodular inner surface, calcified or not, and with a width of at least 2 mm. Interstitial changes compatible with asbestosis was assessed from the HRCT interstitial abnormalities that persisted in prone position. Radiological abnormalities namely interstitial changes compatible with asbestosis, pleural plaques and diffuse pleural thickening, were independently rated (no, possible, certain) by three readers from a five-reader panel who were blinded to the subject's medical data and occupational characteristics. The median of the three rates was retained as the final rate. Only certain pleural plaques and interstitial changes compatible with asbestosis were considered for statistical analyses due to the small number of cases of pleural diffuse thickening.

### Statistical methods

The prevalence was analyzed using a standard logistic model. Such a model fits a linear model on the logistic transformation of the prevalence.

In mathematical terms, if we denote by P the prevalence, the fitted model is:

log⁡(P(1−P))=α+β1X1+β2X2

exp(β_1_) (respectively exp(β_2_)) is the Odds Ratio by unit of X_1 _(respectively X_2_), that is the number by which the disease odds is multiplied if exposure X_1 _(respectively X_2_) increases by one unit. Different models are computed with different exposure variables: time since first exposure, total cumulative exposure, duration of exposure and mean exposure, that is the total cumulative exposure divided by the duration of exposure. The retained models discard the variables which are non-significant once adjusted on the others. The overall fit of the data was assessed using the Akaike Information Criterion (AIC), the better fit being indicated by higher values of the AIC.

From each of the selected model, a prediction of the population prevalence can be obtained for each given asbestos values of the exposure variables.

A second analysis tries to infer the effect of the asbestos exposure on the incidence of pleural plaques based on the model developed by Järvholm [5] which we extend by incorporating the quantitative exposure assessment obtained in the present study. This model presupposes that the probability of belonging to this study population does not depend on the disease status (given the exposure), and that the exposure has stopped at the time of data collection. Assuming that the incidence is a power function of a lagged time since first exposure multiplied by a function of the exposure (either cumulative exposure and/or mean exposure and/or duration of exposure or the log transformed versions of these variables), it can be identified from the prevalence data (see appendix). The different (non-nested) models we compared using the Akaike information criterion.

## Results

Table 1 presents the age and smoking structure as well as all collected variables with respect to the asbestos exposure, according to the diagnosis of the CT-scan among the 1011 subjects included in this study. As expected, subjects with pleural plaques or asbestosis are older than the healthy population. We note that while the numbers of never smokers are lower among the cases, the number of pack years is similar in the three groups.

**Table 1:** Asbestos exposure and confounders among males according to disease status

	**Healthy subjects (n = 476)**		**Pleural plaques (n = 474)**		**Fibrosis (n = 61)**	
age	60.6	±8.0	64.0	±8.2	64.8	±9.2
Non-smokers (n(%))	156	32.8%	116	24.5%	18	23.1%
Ex-smokers (n(%))	244	51.3%	301	63.5%	47	60.3%
Pack-years	21.7	±19.2	20.2	±16.8	21.2	±19.6
Smokers (n(%))	76	16.0%	57	12.0%	13	16.7%
Pack-years	29.7	±24.3	25.7	±20.7	26.2	±26.7
Time since first Exposure (y)	34.3	±8.6	39.5	±9.2	38.5	±9.8
Age at first Exposure (y)	26.3	±9.0	24.6	±7.9	26.3	±8.3
Duration of Exposure (y)	22.8	±9.0	25.1	±9.4	23.4	±9.6
Cumulative Exposure (y.f/ml)	88.9	±92.4	137.0	140.8	143.3	±135.4
Mean Exposure (f/L)	3.9	±3.4	5.5	±5.1	6.5	±5.6

Results are given as means and standard deviations or as numbers and percent, as relevant.

All asbestos exposure indices, except age at first exposure, are larger when either pleural plaques or asbestosis have been diagnosed. It is noteworthy that the differences in time since first exposure correspond approximately to the age differences between the groups.

Table 2 presents two alternative logistic models for the prevalence of pleural plaques as a function respectively of the mean asbestos exposure level and the cumulative exposure. These models were obtained by successively including all asbestos variables and several potential confounders (age, obesity, period of birth). None of the latter confounding variables reached a 10% significance levels, so that they were not entered in the model.

**Table 2:** Modelling the prevalence of pleural plaques using logistic regression

	Model 1			Model 2		
	OR	95%CI	p	OR	95%CI	p

Time since first exposure (y)	1.075	1.059, 1.092	<0.001	1.067	1.051, 1.083	<0.001
Mean exposure (f/ml)	1.114	1.077, 1.153	<0.001			
Cumulative exposure (10 y.f/ml)				1.036	1.023, 1.049	<0.001
Akaike Information Criterion	1228.0			1235.4		
Model: Log(P/1-P)=	-3.22+0.072 TSFE (y) +0.108 mean expo (f/ml)			-2.83+0.065 TSFE (y) +0.0035 cum expo (y.f/ml)		

OR: Odds Ratio by exposure unit

95%CI: 95% Confidence Intervals

p: p-value of a test of a zero coefficient

P: Prevalence of pleural plaques.

As indicated by the higher value of the AIC, the model including TSFE and the mean asbestos exposure showed a better fit than the model including TSFE and cumulative exposure. It is notable that duration of exposure was not a significant predictor of the prevalence of pleural plaques after adjusting for TSFE. For subjects with identical exposure levels and TSFE, the pleural plaques prevalence was even slightly lower for subjects with longer duration of exposure.

The models were also fitted within the two recruitment subpopulations and to the subpopulation of all subjects with a CEI less than 250 fibers/ml.years. All results were compatible with the models presented in Table 2.

**Figure 1:**
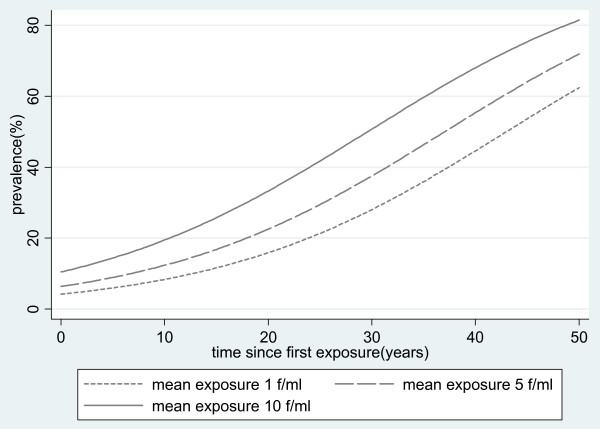
Fitted prevalence of pleural plaques according to time since first exposure and mean asbestos exposure.

Model 1 predicts e.g. that the prevalence is multiplied by 1.075, or equivalently increases by 7.5%, with each year after the onset of exposure. Figure 1 shows the predicted evolution of the prevalence graphically for selected values of the mean exposure.

When estimating an incidence, using a complementary log-log regression, the models with the best fit included linear exposure concentration and/or cumulative exposure rather than log-transformed variables. As for the logistic model the duration of exposure did not significantly contribute to the fit and was dropped. With respect to the minimum latency time, all models with a minimum latency between 6 and 12 years yielded a virtually identical fit.

Table 3 presents the fit of the complementary log-log regression with a minimal latency of 10 years and its interpretation as a model for the incidence of pleural plaques.

**Table 3:** Modelling the incidence of pleural plaques using a complementary log-log regression

	Model 1			Model 2		
	coefficient	95%CI	p	coefficient	95%CI	p

Intercept Log(k/(α +1))	-5.093	-6.098, -4.089	<0.001	-4.553	-5.519, -3.588	<0.001
Log time since first exposure	1.340	1.045, 1.635	<0.001	1.201	0.913, 1.490	<0.001
Mean exposure (f/ml)	0.069	0.049, 0.090	<0.001			
Cumulative exposure (100 y.f/ml)				0.211	0.142, 0.281	<0.001
Akaike Information Criterion	1230.3			1239.4		
Fitted Incidence model	0.00823 × (TSFE-10)^0.340 ^× exp(0.069. mean expo)			0.0127 × (TSFE-10)^0.201 ^× exp(0.00211.cum expo)		

95%CI: 95% Confidence Intervals

p: p-value of a test of a zero coefficient

TSFE: Time Since First Exposure.

The parameters of the incidence curve can be interpreted in terms of absolute and relative risks. E.g the yearly incidence rate for 2 f/ml and 20 years since first exposure is estimated at 0.00823 · 10^0.340 ^· exp(0.0690 · 2) = 2.1%. Similarly according to model 1, the relative risk of a subject with a 10 f/ml mean exposure compared with a 2 f/ml mean exposure is exp(0.069 · (10-2)) = 1.74. The minimum observed latency for a subject with pleural plaques was 16 years. We can note that the fit of the logistic model to the data as assessed by the Akaike information criterion, seems to be very close to the complementary log-log model.

Finally, Table 4 presents the best-fitting logistic models of the prevalence of asbestosis. Although the coefficients of a model including time since first exposure and cumulative exposure are statistically significant, the best fitting model according to the Akaike information criterion includes age and mean exposure.

**Table 4:** Modelling the prevalence of asbestosis using logistic regression

	Model 1			Model 2		
	OR	95%CI	p	OR	95%CI	p

Time since first exposure (y)	1.03	1.00, 1.06	0.038			
Mean exposure (f/ml)				1.09	1.04, 1.14	<0.001
Cumulative exposure (10 y.f/ml)	1.03	1.01, 1.04	0.001			
Age (y)				1.07	1.03, 1.11	<0.001
Akaike Information Criterion	446.0			427.9		
Model: Log(P/1-P)=	-4.22+0.031 TSFE(y) +0.0027 cum expo (y.f/ml)			-7.55+ 0.088 mean expo (f/ml) +0.068 age(y)		

OR: Odds Ratio by exposure unit

95%CI: 95% Confidence Intervals

p: p-value of a test of a zero coefficient

P: Prevalence of asbestosis

TSFE: Time Since First Exposure.

When fitting a complementary log-log regression, some coefficients were negative, indicating that some hypotheses on which this model is based are not met.

## Discussion

Overall, TSFE was the key variable for both pleural plaques and asbestosis, and duration of exposure was not found to be predictive, adjusted on TSFE High exposure concentration to asbestos appears also to be a significant variable with a less significant than TSFE. Regarding these results, clinicians should mainly consider these two variables before including exposed subjects in a CT Scan screening. According to our modelling of incidence, a useful periodicity of survey of pleural plaques by CT-scan may range between 5 to 10 years

This population is characterized by an asbestos exposure assessment based mainly on historical measures and the systematic use of CT-scan for the diagnosis of asbestos-related diseases. To our knowledge, it is the first time that such data are published.

Most of published studies reported a positive dose-response relationship between prevalence of pleural plaques and asbestos exposure [1,17]. Time since first exposure is known to be strongly associated with pleural plaques in the literature [7]. Jakobsson et al. [18] reported that time since first exposure was more correlated to prevalence of pleural plaques assessed on chest X-rays than cumulative exposure, after adjustment on age and smoking status. More recently, Koskinen et al. described a prevalence of bilateral pleural plaques ranging between 1.2% (latency < 16 years) to 32.2% (latency > = 40 years) in a large study of construction, shipyard and asbestos industry workers [3]. In this study, a lesser positive correlation with the duration of asbestos exposure was also reported. Erhlich at al. [19] showed in multiple regression analysis that only time since first exposure was significantly related to prevalence of pleural plaques, but neither cumulative exposure nor duration of exposure. Our results however showed that both mean (or cumulative) exposure and time since first exposure are determinants for the prevalence. This discrepancy may be explained by the better precision of our exposure data.

Asbestosis is also associated with latency [3] but the main parameter seems to be cumulative exposure [18-20]. A threshold was estimated about 25 fibres/ml.years [21] that confirmed experimental and epidemiological data. However, some cases were described below this threshold, mainly in case of very intense exposure [19]. It is generally recognized that the latency of asbestosis is shorter with increasing intensity of exposure [22]. Again, we found that the time since first exposure and cumulative exposure are independent factors of the prevalence of asbestosis. According to our results, we propose to define subjects at high risk of developing asbestos-related diseases on the basis of time since first exposure and high level of exposure.

The question of the optimal periodicity of CT-scan follow-ups in asbestos workers remains unsolved. In our study, no estimates could obtain for the incidence of asbestosis, but incidence model of pleural plaques shows that the increase is modest over the years. For example, according to the model including only time since first exposure and mean exposure, the yearly incidence increases from 0.8% 10 years after the onset of exposure to 2.4% 30 years after exposure onset for a mean exposure of 1 fibre/ml. This finding agrees with the literature, even if only few papers were published on this topic. In a cohort survey (> 20 years) of insulators, the incidence of pleural plaques was estimated between 15% to 21% and between 4 to 10% for parenchymal abnormalities [19]. According to our findings, a useful periodicity of medical survey by CT-scan for pleural plaques may range between 5 to 10 years (4% to 24% of new cases of pleural plaques accordingly to our data), that it is less frequent that the recent statement of the ATS [1].

However, some limits of our study have to be discussed.

First, the population used in this study is not representative of the general population as only asbestos-exposed volunteers were included. We included subjects from several plants and the participation rates were not assessed for all of them, reason why we do not perform analyses by industrial sectors. Moreover, we enrolled only supposed healthy subjects for the screening results, which entailed a selection of these subjects. E.g., we can assume that the most severe cases of asbestosis in these populations were diagnosed before inclusion on a clinical basis and were thus not included in the study population. This, as well as the small number of cases, may probably explain the difficulties to fit the data and obtain a valid model for the asbestosis incidence. However, if this selection may influence the parameter values in the model of estimated prevalence for asbestosis in our sample, this selection bias is probably minor for pleural plaques as this disease is usually asymptomatic [23].

A second point is the availability of dose exposure assessment and accordingly the usefulness of our results for clinicians. Atmospheric measurements, as in our study, are rarely available and retrospective assessment of asbestos exposure is usually based on job-specific questionnaires and job exposures matrices [24]. None of these two methods is clearly the better [25]. However, some combinations of the two may provide sufficiently accurate estimates of asbestos exposure [26] and may be use d in actual clinical practice.

Another point is the technique of HRCT used in this program. We have already discussed in a previous publication [13] that a 6 slices protocol is considered adequate for the diagnosis of asbestosis and had been recommended [2]. For pleural plaques, the use of mediastinal windows and a continuous acquisition of data minimized the risk of missing pleural abnormalities.

Two basically equivalent statistical models were used to model the prevalence of pleural plaques and asbestosis in this population. However, fitting this model on these data allowed us, given some assumptions, to get a mathematically simple expression for the incidence. The most important assumption is that the possibility of being observed (i.e. being included in the population) does not depend on the presence of pleural plaques, respectively asbestosis. It has already been discussed that this does not seem to be a problem for pleural plaques but certainly is one for asbestosis, hence the inconsistent results for its interpretation. The second assumption is that the asbestos exposure does not vary with time for each subject when the prevalence was assessed. As most workers are retired and as asbestos is now banned, this does not appear to be a major issue. The last assumption is that the incidence curve takes a specific functional form which states that the incidence is zero until a given minimum latency and increases as a power function of (time since first exposure minus minimum latency), multiplied by a basic incidence depending only on the past asbestos exposure.

## Conclusion

Time since first exposure and mean (or cumulative) exposure to asbestos were both independently and significantly associated to the prevalence and, to the incidence of pleural plaques and asbestosis. These two parameters have to be included in definitions of high risk subjects for non malignant asbestos related diseases. If the evaluation of the lung cancer screening procedures by Low-Dose CT-Scan confirms his effectiveness, it will be necessary to narrow the definition of people suitable for such screening programs. Among existing models of lung cancer prediction, no one included asbestos exposure to our knowledge. Our results indicate that both latency and dose should be discussed if these prediction models are to consider asbestos exposure. Finally, short-periodicity of survey of pleural plaques by CT-Scan seemed not to be warranted.

## Competing interests

The authors declare that they have no competing interests.

## Authors' contributions

CP conceived of the study, participated in its design and coordination, interpretation of data and wrote the manuscript.

AM participated to the statistical analyses and the interpretation of data.

PW performed the statistical analyses, provided support in the interpretation of data, and manuscript writing.

ML participated in the design and coordination of the study, and provided support in the

interpretation of data, and manuscript writing.

All authors have read and approved the final version of the manuscript._

## Appendix A

### Inferring an incidence model based on prevalence data: the mathematical model

This derivation follows the mathematical model proposed by Järvholm (1992)

Let us assume that the incidence model has following mathematical form

(1) I(t) = k.(t-w)^α^.f^β^.d^γ ^   if t>w

The constant k may depend on other characteristics like smoking or age at first exposure, but is not allowed to depend on time. The latter condition is not an intrinsic assumption, but the model cannot be fitted using standard software unless it is met.

This means in particular that while age cannot be fitted but the period of birth is allowed.

This has also the consequence that this model may only be applied assuming that the exposure has ceased at the time of the disease assessment by TDM.

We postulate the existence of a cohort exposed to asbestos, for which each subject was submitted to a TDM at some time t since his/her first exposure to asbestos.

We assume further that the fact that this disease assessment was independent of the actual disease status.

Let us further denote by N(t), the number of exposed subjects in this cohort at time t and P(t) the disease prevalence (i.e. the prevalence of pleural plaques) at this time.

At time t, N(t)(1-P(t)) subjects do not show pleural plaques and are therefore at risk of developing pleural plaques.

In a Δt time span following t, the number of new cases can be written as N(t).ΔP(t) on one side, and, if Δt is a short time span, as the Δt.I(t).N(t)(1-P(t)) on the other side.

Thus we have N(t).ΔP(t) = Δt.I(t).N(t)(1-P(t))

Cancelling out N(t) and dividing both terms by Δt, we get

$$\frac{\Delta (1-P(t))}{\Delta t}=(1-P(t))I(t)$$

Letting Δt go to zero and solving the differential equation, we obtain

$$\ln(1-P(t))=\int -I(u)du=-k.{\text{f}}^{\beta }.d^{\gamma }\int_{w}^{t}{\left(u-w\right)}^{\alpha }du=\frac{-k}{\alpha +1}.{\text{f}}^{\beta }.d^{\gamma }.{\left(t-w\right)}^{\alpha +1}$$

Taking the natural logarithm of the opposite, we obtain following formula

$$\ln(-\ln(1-P(t)))=\ln(\frac{k}{\alpha +1})+(\alpha +1)\ln(t-w)+\beta .\ln(f)+\gamma .\ln(d)$$

This is the formula for a complementary log-log regression of the prevalence data which is easily fitted with any software (e.g. SAS, Stata, GLIM) providing a generalized linear modelling framework. The independent variables fitted are, in this case, the logarithms of the exposure variables and the logarithm of the lagged time since first exposure.

When the fitted independent exposure variables consist in a single untransformed exposure concentration f, the incidence is

I(t) = k.(t-w)^α^.exp(βf)   if t>w

## References

[B1] American Thoracic Society (2004). Diagnosis and initial management of nonmalignant diseases related to asbestos. Am J Respir Crit Care Med.

[B2] Consensus Report (1997). Asbestos, asbestosis, and cancer: the Helsinki criteria for diagnosis and attribution. Scandinavian journal of work, environment & health.

[B3] Koskinen K, Zitting A, Tossavainen A, Rinne JP, Roto P, Kivekas J, Reijula K, Huuskonen MS (1998). Radiographic abnormalities among Finnish construction, shipyard and asbestos industry workers. Scandinavian journal of work, environment & health.

[B4] Tossavainen A (2000). International expert meeting on new advances in the radiology and screening of asbestos-related diseases. Scandinavian journal of work, environment & health.

[B5] Jarvholm B (1992). Pleural plaques and exposure to asbestos: a mathematical model. International journal of epidemiology.

[B6] Stayner L, Smith R, Bailer J, Gilbert S, Steenland K, Dement J, Brown D, Lemen R (1997). Exposure-response analysis of risk of respiratory disease associated with occupational exposure to chrysotile asbestos. Occupational and environmental medicine.

[B7] Boffetta P (1998). Health effects of asbestos exposure in humans: a quantitative assessment. La Medicina del lavoro.

[B8] Begin R, Christman JW (2001). Detailed occupational history: the cornerstone in diagnosis of asbestos-related lung disease. American journal of respiratory and critical care medicine.

[B9] Das M, Muhlenbruch G, Mahnken AH, Hering KG, Sirbu H, Zschiesche W, Knoll L, Felten MK, Kraus T, Gunther RW, Wildberger JE (2007). sbestos Surveillance Program Aachen (ASPA): initial results from baseline screening for lung cancer in asbestos-exposed high-risk individuals using low-dose multidetector-row CT. Eur Radiol.

[B10] Callol L, Roig F, Cuevas A, de Granda JI, Villegas F, Jareno J, Arias E, Albiach JM (2007). Low-dose CT: a useful and accessible tool for the early diagnosis of lung cancer in selected populations. Lung cancer (Amsterdam, Netherlands).

[B11] Fasola G, Belvedere O, Aita M, Zanin T, Follador A, Cassetti P, Meduri S, De Pangher V, Pignata G, Rosolen V, Barbone F, Grossi F (2007). Low-dose computed tomography screening for lung cancer and pleural mesothelioma in an asbestos-exposed population: baseline results of a prospective, nonrandomized feasibility trial – an Alpe-adria Thoracic Oncology Multidisciplinary Group Study (ATOM 002). The oncologist.

[B12] Paris C, Galateau-Salle F, Creveuil C, Morello R, Raffaelli C, Gillon JC, Billon-Galland MA, Pairon JC, Chevreau L, Letourneux M (2002). Asbestos bodies in the sputum of asbestos workers: correlation with occupational exposure. Eur Respir J.

[B13] Paris C, Benichou J, Raffaelli C, Genevois A, Fournier L, Menard G, Broessel N, Ameille J, Brochard P, Gillon JC, Gislard A, Letourneux M (2004). Factors associated with early-stage pulmonary fibrosis as determined by high-resolution computed tomography among persons occupationally exposed to asbestos. Scandinavian journal of work, environment & health.

[B14] Imbernon E, Goldberg M, Spyckerell Y, Steinmetz J, Bonenfant S, Fournier B (2004). Use of a job-exposure matrix for the screening of occupational exposure to asbestos. Revue d'epidemiologie et de sante publique.

[B15] Aberle DR, Gamsu G, Ray CS (1988). High-resolution CT of benign asbestos-related diseases: clinical and radiographic correlation. Ajr.

[B16] Gevenois PA, de Maertelaer V, Madani A, Winant C, Sergent G, De Vuyst P (1998). Asbestosis, pleural plaques and diffuse pleural thickening: three distinct benign responses to asbestos exposure. Eur Respir J.

[B17] Hillerdal G, Loddenkemper R, Antony VB (2002). Asbestos-related pleural disease including diffuse malignant mesothelioma. Pleural diseases.

[B18] Jakobsson K, Stromberg U, Albin M, Welinder H, Hagmar L (1995). Radiological changes in asbestos cement workers. Occup Environ Med.

[B19] Ehrlich R, Lilis R, Chan E, Nicholson WJ, Selikoff IJ (1992). Long term radiological effects of short term exposure to amosite asbestos among factory workers. British journal of industrial medicine.

[B20] Becklake MR (1991). Asbestos and other fiber-related diseases of the lungs and pleura. Distribution and determinants in exposed populations. Chest.

[B21] Doll R, ed (1985). Effects on health of exposure to asbestos. Health and Safety Executive.

[B22] Sluis-Cremer GK, Hnizdo E (1989). Progression of irregular opacities in asbestos miners. British journal of industrial medicine.

[B23] Chapman SJ, Cookson WO, Musk AW, Lee YC (2003). Benign asbestos pleural diseases. Current opinion in pulmonary medicine.

[B24] Ahrens W, Jockel KH, Brochard P, Bolm-Audorff U, Grossgarten K, Iwatsubo Y, Orlowski E, Pohlabeln H, Berrino F (1993). Retrospective assessment of asbestos exposure – I. Case-control analysis in a study of lung cancer: efficiency of job-specific questionnaires and job exposure matrices. International journal of epidemiology.

[B25] Orlowski E, Pohlabeln H, Berrino F, Ahrens W, Bolm-Audorff U, Grossgarten K, Iwatsubo Y, Jockel KH, Brochard P (1993). Retrospective assessment of asbestos exposure – II. At the job level: complementarity of job-specific questionnaire and job exposure matrices. International journal of epidemiology.

[B26] Nam JM, Rice C, Gail MH (2005). Comparison of asbestos exposure assessments by next-of-kin respondents, by an occupational hygienist, and by a job-exposure matrix from the National Occupational Hazard Survey. American journal of industrial medicine.

